# Nucleus Basalis of Meynert Volume and Cognitive Impairment in Parkinson’s Disease Before and After Deep Brain Stimulation of the Subthalamic Nucleus

**DOI:** 10.3390/brainsci15060630

**Published:** 2025-06-11

**Authors:** Vibuthi Sisodia, Yarit Wiggerts, Anouk A. Boogaard, Arthur W. G. Buijink, Rozemarije A. Holewijn, Bart E. K. S. Swinnen, Gert J. Geurtsen, Rick Schuurman, Rob M. A. de Bie

**Affiliations:** 1 Department of Neurology, Amsterdam UMC, Amsterdam Neuroscience, University of Amsterdam, Meibergdreef 9, 1105 AZ Amsterdam, The Netherlands; 2 Department of Neurosurgery, Amsterdam UMC, Amsterdam Neuroscience, University of Amsterdam, Meibergdreef 9, 1105 AZ Amsterdam, The Netherlands; 3 Department of Neurology, University Hospitals Leuven, Herestraat 49, 3000 Leuven, Belgium; 4 Department of Medical Psychology, Amsterdam UMC, Amsterdam Neuroscience, University of Amsterdam, Meibergdreef 9, 1105 AZ Amsterdam, The Netherlands

**Keywords:** Parkinson’s disease, deep brain stimulation, cognition, nucleus basalis of Meynert

## Abstract

**Objectives:** To investigate whether a smaller volume of the nucleus basalis of Meynert (NBM) is associated with (1) preoperative cognitive impairment and (2) cognitive decline six months after deep brain stimulation (DBS) of the subthalamic nucleus (STN) in patients with Parkinson’s disease (PD), using detailed neuropsychological assessment (NPA). **Methods**: PD patients from the GALAXY and DBS-MODE study were included if they had a preoperative MRI and NPA. NBM volume was measured using voxel-based morphometry. Regression analyses were conducted to assess associations between NBM volume and (1) global NPA scores at baseline, (2) baseline cognitive categories (i.e., normal cognition, mild cognitive impairment (MCI) and Parkinson’s disease dementia (PDD)), (3) change in NPA score and (4) cognitive decline based on the reliability change index six months after STN DBS. **Results**: For 129 patients, NBM volume was significantly associated with baseline cognitive categories (χ^2^ = 42.4, *p* < 0.001). However, smaller NBM volume appeared to be associated only with PDD (B = −2.2, *p* = 0.036), not with MCI. In 103 patients, no association was found between NBM volume and NPA score at baseline, change in NPA score nor cognitive decline six months after STN DBS. **Conclusions**: Smaller NBM volume appears to be associated with PDD at baseline, but not with cognitive decline six months after STN DBS.

## 1. Introduction

Cognitive impairment is a frequent non-motor symptom in patients with Parkinson’s disease (PD) [1,2,3,4]. Within five years of diagnosis, 40–50% of patients meet the criteria for mild cognitive impairment (PD-MCI), and 10–20% meet the criteria for Parkinson’s disease dementia (PDD) [1,2,4]. As the disease progresses, the prevalence of PDD increases, affecting approximately 50% of patients a decade after diagnosis [2]. These cognitive impairments have profound effects on quality of life and activities of daily living [5,6,7]. However, the underlying mechanisms remain unclear. Cholinergic pathways are believed to play a crucial role in cognitive function, with acetylcholine depletion being associated with cognitive impairment [8,9]. The nucleus basalis of Meynert (NBM) serves as a critical source of cortical acetylcholine and the accumulation of Lewy bodies in the NBM may lead to the depletion of cholinergic neurotransmission [9,10]. Histological and positron emission tomography (PET) studies have reported significant cortical cholinergic deficits in PD patients with cognitive impairment [11]. Inconsistent results have been reported regarding the association between NBM degeneration based on Magnetic Resonance Imaging (MRI) and cognitive impairment in PD [12]. Notably, cognition was often assessed with global screening tools or a limited cognitive test battery [12].

Deep brain stimulation (DBS) of the subthalamic nucleus (STN) is an established treatment for the motor symptoms of PD, improving motor function and quality of life substantially [13,14,15]. However, its impact on cognitive function remains less clear. While a transient decline in verbal fluency has been reported following DBS, findings regarding other cognitive domains are inconsistent [16,17,18,19]. Currently, it remains unclear which patients are at greater risk of developing cognitive impairment after DBS treatment [20]. Kübler et al. found that structural degeneration of the NBM predicted cognitive decline in PD patients after DBS treatment, suggesting that structural vulnerability in cholinergic pathways may exacerbate postoperative cognitive changes [21]. However, cognition was assessed with the Mini-Mental State Examination (MMSE), while a comprehensive neuropsychological assessment (NPA) provides a more in-depth and accurate evaluation of cognitive decline in PD patients [21,22]. The present study aims to determine whether a smaller NBM volume in PD is associated with (1) preoperative cognitive impairment and (2) cognitive decline six months after STN DBS treatment by integrating neuroimaging and detailed NPA in the cohorts of the GALAXY trial and DBS-MODE trial [23,24].

## 2. Materials and Methods

### 2.1. Study Participants

Data for this study were retrieved from the GALAXY trial and DBS-MODE trial. In the GALAXY trial, PD patients were randomized to either general anesthesia or local anesthesia for bilateral STN DBS surgery. The study protocol and results of the GALAXY trial have previously been published [24,25]. The DBS-MODE trial is an ongoing randomized controlled trial investigating the safety and efficacy of STN DBS for the treatment of motor symptoms in patients with PDD. The study protocol has previously been published [23]. For evaluating the association between NBM volume and preoperative cognition, participants were included in the analysis if they had a preoperative MRI scan and NPA. For evaluating the association between NBM volume and cognitive decline after STN DBS, participants were included in the analysis if they had a preoperative MRI scan and NPA at both baseline and 6-month follow-up. Participants were excluded if four or more cognitive tests were missing in the NPA.

### 2.2. Neuropsychological Assessment

The NPA was conducted at baseline in the ON-drug phase in both the Galaxy and DBS-MODE study. The 6-month follow-up NPA was only conducted in the GALAXY trial and was performed during the ON-drug phase and DBS ON condition. The following cognitive domains and tests were included in the NPA: language (Boston naming test (BNT) and Wechsler Adult Intelligence Scale IV (WAIS)-Similarities), memory (Rey Auditory Verbal Learning Test (RAVLT) and Rivermead Behavioural Memory Test stories (RBMT)), attention (Trail Making Test (TMT)-A, the Stroop Color-Word Test (Stroop)-I), executive function (TMT-B and Stroop-III), and visuospatial function (Judgement of Line Orientation (JOLO) and clock drawing) [26,27,28,29,30,31,32,33,34].

A global NPA score at both assessments was calculated using ANDI-NORMS. ANDI-NORMS is an extensive Dutch neuropsychological normative database that provides demographically corrected reference values based on data from over 26,000 healthy individuals [35]. The database adjusts for age, sex, and education, thereby allowing for precise estimation of individual cognitive performance relative to healthy controls. The global NPA score was derived through a multivariate statistical analysis correcting for test-intercorrelations by comparing each patient’s cognitive test z-scores to corresponding normative values, representing the overall cognitive performance. However, WAIS-IV Similarities and JOLO are not included in ANDI-NORMS and therefore could not be incorporated into the global NPA score. The change in the global NPA score was calculated as the difference in NPA score between baseline and six-month follow-up after STN DBS.

The baseline cognitive categorizations were normal cognition, PD-MCI and PDD. PD-MCI was defined using the current diagnostic criteria: a deficit in two tests within one cognitive domain or in one test across two cognitive domains, without significant impairment in daily functioning due to cognitive complaints [36]. PDD diagnosis was based on the diagnostic criteria published in 2007: a deficit in two tests in at least two cognitive domains, while cognitive symptoms impair functioning in daily life [37]. A threshold of 1.5 and 2.0 standard deviation (SD) below appropriate norms was used to identify deviating cognitive test scores for PD-MCI and PDD, respectively. The ANDI-NORMS database was employed to assess each patient’s test scores [35]. The WAIS-IV Similarities and JOLO were evaluated separately. The baseline cognitive status of the patient was determined based on all tests. Clinically relevant cognitive decline was defined as a decline on three or more cognitive tests in the NPA, based on a Reliable Change Index (RCI) of −1.645 or lower in at least two domains at the 6-month follow-up compared to baseline using the aforementioned normative scores [38].

### 2.3. Imaging

Preoperative high-resolution anatomical T1 3D Turbo Field Echo (TFE) MRI scans (echo time 4.0 ms, repetition time 8.8 ms, flip angle 8°, field of view 256 × 256 mm, voxel size 0.5 × 0.5 × 0.9 mm, number of slices 189) were obtained using three 3.0 Tesla Philips Ingenia scanners of the exact same model (Philips Healthcare, Best, The Netherlands) and processed for volumetric analysis. The NBM volume was first assessed through voxel-based morphometry with the CAT12 toolbox, an extension of SPM12 in MATLAB (2021a). Image preprocessing involved skull-stripping, segmentation of gray matter, and spatial normalization to Montreal Neurological Institute (MNI) space. The NBM mask was derived from the probabilistic anatomical maps developed by Zaborszky et al., as implemented in the Anatomy Toolbox (version 2.2) [39]. These maps are based on histological delineations of the cholinergic basal forebrain nuclei in post-mortem human brains, with the Ch4 region corresponding to the NBM. To account for interindividual differences in brain size, which are influenced by age and sex, NBM volumes were normalized to the total intracranial volume (TIV). The following formula was used to calculate the normalized NBM volume: (NBM volume in mm^3^/TIV in mm^3^)∗10,000.

For a second calculation of the NBM volumes, Brainlab (Brainlab AG, Munich, Germany) was used. Within Brainlab, the Image Fusion Element was used to linearly co-register the T1 and T2 scans. The NBM structures were automatically segmented in 3D per patient and hemisphere separately (based on patient-specific anatomy) using Elements Object Management. The generated output included the volume of the NBM for the left and right hemispheres separately, which were then summed to obtain the total NBM volume. The location of the segmented NBM was visually inspected by one researcher (VS) to verify its accuracy. In cases of uncertainty, consultation with a neurosurgeon (RS) was possible. As no TIV was provided in Brainlab, the TIV derived from the SPM12 in MATLAB was used to calculate the normalized volume of the NBM obtained from Brainlab using the same formula as before.

### 2.4. Statistical Analysis

All data analyses were performed using SPSS Version 28.0 (IBM Corp, Armonk, NY, USA). The mean difference in the normalized NBM volume obtained from SPM12 and Brainlab was assessed with the paired samples *t*-test. To assess the association between normalized NBM volume and cognition at baseline, multivariate linear regression and multinomial logistic regression were used for the baseline NPA score and baseline cognitive categorization (i.e., normal cognition, PD-MCI or PDD), respectively. Both analyses were adjusted for age, disease duration and MRI scanner. Normal cognition was the reference category in the multinomial logistic regression. To assess the association between NBM volume and cognitive change six months after STN DBS, multivariate linear regression was used for the change in NPA score and multivariate logistic regression for clinically relevant cognitive decline. Both regression analyses were adjusted for age, disease duration, MRI scanner, and PD-MCI at baseline. Lastly, to examine the potential impact of excluding WAIS-IV Similarities and JOLO from the baseline NPA and change in NPA score, sensitivity analyses were performed using multivariate linear regression with a baseline composite T-score and change in composite T-score based on all previously mentioned cognitive tests. The analysis with baseline composite T-score was adjusted for age, disease duration and MRI scanner. The analysis with change in composite T-score was adjusted for age, disease duration, MRI scanner, and PD-MCI at baseline. If only one test was missing in the NPA, the group mean for that specific test was imputed. If two or more tests were missing, imputation was based on the worst-case scenario. *P* values of less than 0.05 were considered statistically significant.

## 3. Results

**Figure 1:**
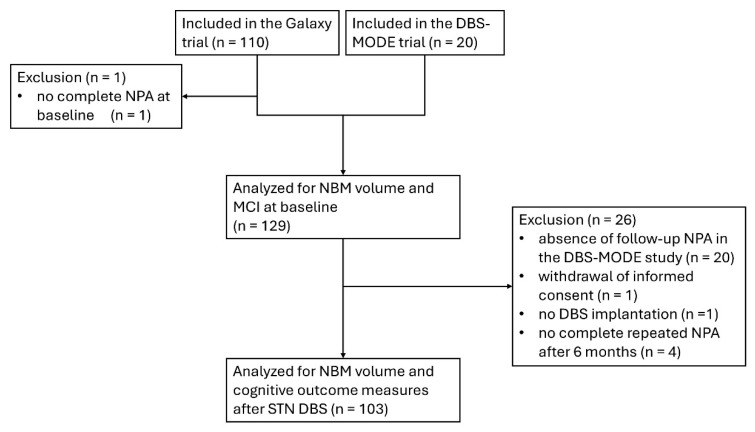
Flowchart of the inclusion of patients in the analysis. DBS, deep brain stimulation; MCI, mild cognitive impairment; NBM, nucleus basalis of Meynert; NPA, neuropsychological assessment; STN, subthalamic nucleus.

For evaluating the association between NBM volume and preoperative cognition, data from 109 patients from the Galaxy trial and 20 from the DBS-MODE trial (a total of 129 patients) were included in the analysis (Figure 1). For the evaluation of NBM volume and cognitive decline six months after STN DBS, data from 103 patients from the Galaxy trial were included. One patient withdrew informed consent, one patient received no DBS implantation, and four patients did not have a complete repeated neuropsychological examination after 6 months of follow-up. The baseline characteristics of the included patients are summarized in Table 1.

### 3.1. Imaging Parameters

The mean NBM volume was 271.7 ± 64.0 mm^3^ using SPM12 and 227.4 ± 41.9 mm^3^ using Brainlab. The mean TIV was 1512.8 ± 176.8 cm^3^. The mean normalized NBM volume was 1.8 ± 0.4 using SPM12 and 1.5 ± 0.2 using Brainlab. The difference in mean normalized NBM volumes between SPM12 and Brainlab was statistically significant with a mean difference of 0.3 (95% CI 0.2 to 0.4, *p* < 0.001).

### 3.2. NBM Volume and Preoperative Cognition

A multivariate linear regression analysis was conducted to examine whether normalized NBM volume, adjusted for age, disease duration and MRI scanner, was associated with the NPA score at baseline. Both the SPM12-derived normalized NBM volume (B = 1.1, 95% confidence interval (CI) −1.6 to 3.8, *p* = 0.416) and the Brainlab-derived normalized NBM volume (B = 4.9, 95% CI −1.2 to 10.9, *p* = 0.116) were not associated with the NPA score at baseline (Table 2).

A multinomial logistic regression analysis was conducted to examine the association between normalized NBM volume and baseline cognitive categories (i.e., normal cognition, PD-MCI and PDD), controlling for age, disease duration and MRI scanner. At baseline, 74 patients were classified with normal cognition, 35 with PD-MCI and 20 with PDD. For the SPM12-derived normalized NBM volume, the regression model was significant (χ^2^ = 42.4, *p* < 0.001) with a Nagelkerke pseudo R^2^ of 0.328. For the comparison between normal cognition and PD-MCI, the normalized NBM volume was not associated with PD-MCI (B = 0.6, 95% CI −0.4 to 1.6, *p* = 0.251). However, for the comparison between normal cognition and PDD, the normalized NBM volume was significantly associated with PDD (B = −2.2, 95% CI -4.9 to 0.1, *p* = 0.036) with an odds ratio of 0.116 (95% CI 0.015 to 0.864) (i.e., the odds of PDD decrease with increasing normalized NBM volume). For the Brainlab-derived normalized NBM volume, the model was also significant (χ^2^ = 50.9, *p* < 0.001) with a Nagelkerke pseudo R^2^ value of 0.826. The normalized NBM volume was not associated with PD-MCI (B = 2.5, 95% CI −0.2 to 5.2, *p* = 0.065). However, for the comparison of normal cognition with PDD, the normalized NBM volume was significantly associated with PDD (B = −4.8, 95% CI −13.8 to −1.4, *p* = 0.006) with an odds ratio of 0.007 (95% CI 0.0 to 0.2).

### 3.3. NBM Volume and Cognitive Decline Six Months After STN DBS

A multivariate linear regression analysis was conducted to examine whether normalized NBM volume, adjusted for age, disease duration, MRI scanner and PD-MCI at baseline, was associated with the change in NPA score six months after STN DBS. Both the SPM12-derived normalized NBM volume (B = −0.4, 95% CI −1.8 to 1.1, *p* = 0.615) and the Brainlab-derived normalized NBM volume (B = −1.3, 95% CI −5.2 to 2.6, *p* = 0.515) were not associated with change in NPA score after STN DBS (Table 3).

A binomial logistic regression analysis was conducted to examine the association between NBM volume and cognitive decline six months after STN DBS, controlling for age, disease duration, MRI scanner and PD-MCI at baseline. In total, 12 patients with cognitive decline and 91 with stable cognition were included. Neither the SPM12-derived normalized NBM volume (B = −1.3, 95% CI −0.4 to 2.9, *p* = 0.141) nor the Brainlab-derived normalized NBM volume (B = −3.9, 95% CI −9.2 to 0.8, *p* = 0.107) was associated with cognitive decline after STN DBS (see Table 3).

### 3.4. Sensitivity Analyses

In the multivariate linear regression analyses, both the SPM12-derived normalized NBM volume (B = 31.2, 95% CI −10.2 to 72.7, *p* = 0.138) and the Brainlab-derived normalized NBM volume (B = 80.5, 95% CI −13.9 to 175.0, *p* = 0.094) were not associated with the baseline composite T-score, after adjusting for age, disease duration and MRI scanner.

In addition, neither the SPM12-derived normalized NBM volume (B = −13.6, 95% CI −32.8 to 5.5, *p* = 0.160) nor the Brainlab-derived normalized NBM volume (B = 20.8, 95% CI −36.2 to 77.7, *p* = 0.472) was associated with the change in composite T-score after adjusting for age, disease duration, MRI scanner and PD-MCI at baseline.

## 4. Discussion

The current study investigated whether a smaller NBM volume is associated with preoperative cognitive impairment and cognitive decline six months after STN DBS in PD. Only PDD at baseline, and not PD-MCI, was associated with a smaller normalized NBM volume. NPA score at baseline was also not associated with normalized NBM volume. Lastly, no statistically significant associations were found between normalized NBM volume and either a change in NPA score or cognitive decline based on the RCI six months after STN DBS. The absence of an association of NBM volume with baseline NPA score, despite a significant association with PDD, suggests that NBM atrophy may be more clearly related to profound and functionally relevant cognitive impairment, rather than MCI or neuropsychological test performance alone. In addition, the relatively small number of patients with PD-MCI and PDD in comparison to patients with normal cognition may also have limited the statistical power needed to detect a significant relationship between NBM volume and NPA score at baseline.

Our findings regarding the association between NBM volume and baseline cognitive categorization are largely in line with previous literature [12]. While no other study has used the NPA score, several have relied on global screening tools or limited cognitive tests. No statistically significant difference in mean NBM volumes has been reported in studies that categorized patients into normal cognition and PD-MCI at baseline [40,41,42,43,44,45]. Only two studies investigated the correlation between NBM volume and cognitive performance in PD patients with normal cognition and PD-MCI and found no statistically significant association between NBM volume and cognition [41,45]. Pereira et al. also assessed NBM volume in patients with stable cognition and PDD. The mean NBM volume was significantly smaller in patients with PDD compared to stable cognition [46]. No regression analysis was performed in this study.

In contrast to our results concerning NBM volume and cognitive decline following STN DBS, Kübler et al. reported that lower NBM volume predicted greater cognitive decline after STN DBS based on MMSE scores [21]. This discrepancy may, in part, be explained by the longer follow-up period in their study (12 months versus 6 months in our cohort), which could allow for more pronounced cognitive changes to emerge. This supports the idea that NBM atrophy may serve as a more robust predictor of cognitive decline over longer time periods, which may primarily be due to the progression of PD rather than a result of DBS. Furthermore, their reported mean NBM volume based on Ch4 (940 mm^3^) differed substantially from those measured in our cohort (272 mm^3^), despite applying the same segmentation method using SPM12. Large differences in NBM volumetry have been reported in the literature with SPM12 and highlight the methodological challenges in reliably quantifying this structure [47]. Of note, the mean normalized NBM volume derived from SPM12 and Brainlab also differed significantly, further illustrating the variability commonly reported in imaging studies of the NBM [47]. Importantly, both measurements in our cohort align with prior findings reported by our research group. [48] In healthy individuals, MRI studies have reported NBM volumes ranging from 40 mm^3^ to 1377 mm^3^, with population means between 300 and 400 mm^3^ [49,50,51,52,53,54,55]. This discrepancy likely reflects methodological differences in anatomical definitions, atlas resolution, and segmentation algorithms between the various tools. NBM volumes derived from MRI have consistently been shown to exceed NBM volumes obtained through histological analysis. Histological studies have reported substantially smaller volumes, ranging from 58.6 to 156 mm^3^, with a mean of 118 mm^3^ [56,57]. This is probably due to an overestimation of MRI-based methods. Because the NBM has diffuse anatomical boundaries and is in close proximity to adjacent structures, it is difficult to accurately delineate the NBM with imaging. This may compromise both the validity and reproducibility of NBM volume measurements and should therefore be carefully considered when interpreting findings from neuroimaging studies involving the NBM. Nevertheless, NBM volumes derived from both SPM12 and Brainlab demonstrated an association between NBM volume and PDD at baseline. Moreover, Kübler et al. did not control for preoperative cognitive impairment using formal diagnostic categories, but instead adjusted for the number of affected cognitive domains [21]. However, this approach is not equivalent to using the PD-MCI and PDD criteria, as these criteria take into account not only the number of impaired cognitive domains but also the severity of deficits and their impact on daily functioning. Furthermore, our study used more sensitive and specific cognitive outcome measures (i.e., NPA score and RCI), which may provide a more nuanced picture of cognitive change compared to the MMSE.

While NBM volume may hold potential as a biomarker for more advanced cognitive impairment, its utility in predicting cognitive decline after STN DBS appears limited in early-stage impairment. In our study cohort, PD-MCI at baseline was associated with cognitive decline after STN DBS. Previous research has also found associations between PD-MCI and accelerated cognitive decline in PD patients without DBS [58,59,60]. Hence, evaluating cognition at baseline could aid clinicians in guiding patients regarding their expected cognitive trajectory during follow-up and explain that the risk of cognitive decline is higher with MCI at baseline, regardless of STN DBS therapy. Future studies should include a comprehensive NPA, examine the association between NBM volume and cognitive domains, adjust for baseline cognitive impairment in regression analyses, and incorporate longer follow-up durations after STN DBS. This would further help to clarify whether postoperative cognitive decline is primarily driven by baseline PD-MCI or whether NBM volume plays an independent role in the cognitive trajectory of PD patients undergoing DBS.

This study systematically evaluated the correlation between NBM volume and cognitive impairment at baseline, including patients with PD-MCI and PDD, as well as cognitive decline six months after STN DBS in PD, using comprehensive NPA data. However, the following limitations need to be mentioned. First, for assessing the association between NBM volume and preoperative cognition, we combined data from two cohorts, which may have introduced confounding factors and sampling bias. However, all participants underwent the same standardized NPA and MRI protocol, and in the regression analyses, we corrected for the use of different MRI scanners. Second, only twelve patients had cognitive decline six months after STN DBS based on the RCI, which may have limited the statistical power to find an association between NBM volume and postoperative cognitive decline. Third, the absence of patients with PDD in the follow-up data limits our ability to generalize findings on cognitive decline after DBS to the full spectrum of cognitive impairment in PD. Fourth, the follow-up period was relatively short, which may be insufficient to capture meaningful changes in cognitive function. At the time of study design of the GALAXY and DBS-MODE study, the potential role of NBM volume in predicting cognitive outcomes was not yet fully anticipated, and extended follow-up for these post-hoc analyses was hence not planned. Other studies evaluating the correlation between NBM volume and cognition during follow-up had longer follow-up periods (i.e., 2 to 5 years) [12]. However, a postoperative follow-up of six months allows for a more accurate assessment of cognitive decline related to DBS, as opposed to the natural progression of PD. Fifth, the ANDI-NORMS database has been validated primarily in Dutch populations, and hence its generalizability to more diverse populations may be limited and should be considered when interpreting the results.

## 5. Conclusions

In this study, we investigated the association between NBM volume with both baseline cognitive impairment and cognitive decline six months after STN DBS in PD. Our results suggest that a smaller NBM volume is associated with PDD at baseline. In addition, the utility of NBM volume in predicting cognitive decline six months after STN DBS appears limited for patients with normal cognition and PD-MCI. Further research is needed with longer follow-up periods to determine whether NBM volume can be used as a reliable predictor of cognitive outcome after DBS.

## Figures and Tables

**Table 1:** Baseline Characteristics.

Characteristic	Total Study Population (*n* = 129)
Age, mean (SD), years	62.1 (8.4)
Age at onset of Parkinson’s disease, mean (SD), years	51.5 (8.9)
Male, no. (%)	89 (69%)
Duration of Parkinson’s disease, mean (SD), years	10.7 (5.0)
On-drug phase Hoehn and Yahr stage, no. (%)	
1	1 (1%)
2	93 (72%)
3	31 (24%)
4	4 (3%)
5	1 (1%)
Levodopa equivalent daily dose, mean (SD)	1544.0 (566.8)
Mattis dementia rating scale, mean (SD)	138.8 (4.7)
PD-CRS, mean (SD)	96.2 (18.1)

PD-CRS, Parkinson’s Disease—Cognitive Rating Scale; SD, standard deviation.

**Table 2:** Normalized NBM volume and preoperative cognition in PD.

Cognitive Assessment	SPM-12 Derived Normalized NBM Volume	Brainlab Derived Normalized NBM Volume
NPA score at baseline	NBM: B = 1.1, *p* = 0.416	NBM: B = 4.9, *p* = 0.116
	Age: B = −0.3, *p* < 0.001 *	Age: B = −0.3, *p* < 0.001 *
	Disease duration: B = 0.1, *p* = 0.555	Disease duration: B = 0.0, *p* = 0.663
	MRI scanner: B = 0.0, *p* = 0.738	MRI scanner: B = 0.0, *p* = 0.817
PD-MCI at baseline ^a^	NBM: B = 0.6, *p* = 0.251	NBM: B = 2.5, *p* = 0.065
	Age: B = 0.0, *p* = 0.182	Age: B = 0.0, *p* = 0.095
	Disease duration: B = 0.0, *p* = 0.094	Disease duration: B = 0.0, *p* = 0.674
	MRI scanner: B = 0.0, *p* = 0.239	MRI scanner: B = 0.0, *p* = 0.300
PDD at baseline ^a^	NBM: B = −2.2, *p* = 0.036 *	NBM: B = −4.8, *p* = 0.006 *
	Age: B = 0.3, *p* < 0.001 *	Age: B = 0.2, *p* < 0.001 *
	Disease duration: B = 0.0, *p* = 0.659	Disease duration: B = 0.0, *p* = 0.870
	MRI scanner: B = 0.0, *p* = 0.847	MRI scanner: B = 0.0, *p* = 0.918

Results are based on analyses of data from 129 patients. MCI, mild cognitive impairment; NBM, nucleus basalis of Meynert; NPA, neuropsychological assessment; PD, Parkinson’s disease; PDD, Parkinson’s disease dementia. Asterisks (*) indicate statistical significance (*p* < 0.05) ^a^ Based on multinomial logistic regression analysis with reference category normal cognition.

**Table 3:** Normalized NBM volume and cognition after STN DBS in PD.

Cognitive Assessment	SPM-12 Derived Normalized NBM Volume	Brainlab Derived Normalized NBM Volume
Change in NPA score	NBM: B = −0.4, *p* = 0.615	NBM: B = −1.3, *p* = 0.515
	Age: B = 0.0, *p* = 0.696	Age: B = 0.0, *p* = 0.589
	Disease duration: B = −0.2, *p* = 0.010 *	Disease duration: B = −0.2, *p* = 0.010 *
	MCI at baseline: B = 1.7, *p* = 0.011 *	MCI at baseline: B = 1.7, *p* = 0.017 *
	MRI scanner: B = 0.0, *p* = 0.189	MRI scanner: B = 0.0, *p* = 0.182
Cognitive decline based on RCI	NBM: B= −1.3, *p* = 0.141	NBM: B = −3.9, *p* = 0.107
	Age: B = 0.0, *p* = 0.519	Age: B = 0.0, *p* = 0.959
	Disease duration: B = −0.1, *p* = 0.118	Disease duration: B = −0.1, *p* = 0.130
	MCI at baseline: B = 1.8, *p* = 0.012 *	MCI at baseline: B = 1.5, *p* = 0.036 *
	MRI scanner: B = 0.0, *p* = 0.958	MRI scanner: B = 0.0, *p* = 0.892

Results are based on analyses of data from 103 patients. Asterisks (*) indicate statistical significance (*p* < 0.05). DBS, deep brain stimulation; MCI, mild cognitive impairment; NBM, nucleus basalis of Meynert; NPA, neuropsychological assessment; PD, Parkinson’s disease; RCI, reliability change index; STN, subthalamic nucleus.

## Data Availability

The data are not publicly available due to privacy and ethical restrictions. The data presented in this study are available on reasonable request from the corresponding author.

## Abbreviations

The following abbreviations are used in this manuscript:

BNT	Boston naming test
CI	Confidence interval
DBS	Deep brain stimulation
JOLO	Judgement of line orientation
MCI	Mild cognitive impairment
MMSE	Mini-Mental State Examination (MMSE)
MNI	Montreal Neurological Institute
MOCA	Montreal Cognitive Assessment
MRI	Magnetic Resonance Imaging
NBM	Nucleus basalis of Meynert
NPA	Neuropsychological assessment
PET	Positron emission tomography
PD	Parkinson’s disease
PDD	Parkinson’s disease dementia
STN	Subthalamic nucleus
TFE	Turbo Field Echo
TIV	Total intracranial volume
TMT	Trail making test
RAVLT	Rey Auditory Verbal Learning Test
RBMT	Rivermead Behavioural Memory Test
RCI	Reliability change index
WAIS	Wechsler Adult Intelligence Scale
